# The Congenital Cataract-Linked G61C Mutation Destabilizes γD-Crystallin and Promotes Non-Native Aggregation

**DOI:** 10.1371/journal.pone.0020564

**Published:** 2011-05-31

**Authors:** Wang Zhang, Hong-Chen Cai, Fei-Feng Li, Yi-Bo Xi, Xu Ma, Yong-Bin Yan

**Affiliations:** 1 State Key Laboratory of Biomembrane and Membrane Biotechnology, School of Life Sciences, Tsinghua University, Beijing, China; 2 Department of Genetics, National Research Institute for Family Planning, Beijing, China; 3 Peking Union Medical College, Tsinghua University, Beijing, China; 4 Institute of Biophysics, Lanzhou University, Lanzhou, China; University of South Florida College of Medicine, United States of America

## Abstract

γD-crystallin is one of the major structural proteins in human eye lens. The solubility and stability of γD-crystallin play a crucial role in maintaining the optical properties of the lens during the life span of an individual. Previous study has shown that the inherited mutation G61C results in autosomal dominant congenital cataract. In this research, we studied the effects of the G61C mutation on γD-crystallin structure, stability and aggregation via biophysical methods. CD, intrinsic and extrinsic fluorescence spectroscopy indicated that the G61C mutation did not affect the native structure of γD-crystallin. The stability of γD-crystallin against heat- or GdnHCl-induced denaturation was significantly decreased by the mutation, while no influence was observed on the acid-induced unfolding. The mutation mainly affected the transition from the native state to the intermediate but not that from the intermediate to the unfolded or aggregated states. At high temperatures, both proteins were able to form aggregates, and the aggregation of the mutant was much more serious than the wild type protein at the same temperature. At body temperature and acidic conditions, the mutant was more prone to form amyloid-like fibrils. The aggregation-prone property of the mutant was not altered by the addition of reductive reagent. These results suggested that the decrease in protein stability followed by aggregation-prone property might be the major cause in the hereditary cataract induced by the G61C mutation.

## Introduction

The vertebrate ocular lens is an avascular tissue composed of ordered fiber cells, and the architecture of the fiber cells provides a structural basis of the optical properties of the lens at the cellular level [1]–[4]. To transmit and focus the light on the retina photoreceptor cells, the lens fiber cells are required to possess the properties of transparency and a high refractive index of ∼1.4. To fulfill these two requirements, the fiber cells are highly differentiated to a unique cellular structure with distinct morphology and composition: the programmed removal of potential light scattering cytoplasmic structures and the expression of fiber cell-specific proteins [5], [6]. The major component of the lens cytoplasmic proteins is crystallins, which are divided into three major classes: α-, β- and γ-crystallins [7]–[9]. The disruption of the protein arrangement or solubility of the crystallins will lead to vision problems including cataract.

The differentiation of the epithelial cells to fiber cells of vertebrates continues throughout the life by laying the young fiber cells surrounded the pre-existing cells, and the oldest interior cells are as old as the individual [1], [10]. The lack of protein degradation systems in the mature fiber cells also requires the crystallins to be long-lived stable proteins since they have to last the whole life span of the individual and face to various stresses [7], [11]. Among the three classes of crystallins, α-crystallin is known to act as a molecular chaperone throughout the development and aging of the lens [12], [13], while β- and γ-crystallins are more likely to be the structural proteins of the lens fiber cells [7], [9], [11]. The significant role of the crystallins in maintaining the optical properties of the lens is evidenced not only by its high expression levels in the fiber cells, but also by cataract caused by aging-related post-translational modifications and familiar inherited mutations [7], [11].

**Figure 1:**
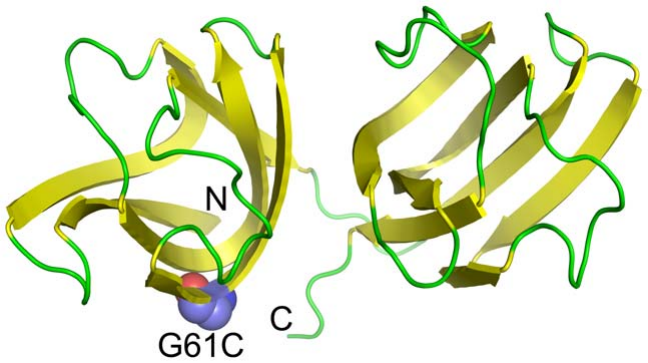
Crystal structure of human γD-crystallin (PDB ID: 1HK0). The position of G61 is highlighted by space filling model. N and C represent the N- and C-terminus of the protein.

In human lens, γC- and γD-crystallins are expressed at the highest level among the γ-crystallins (about 80%) [14]. Consequently, autosomal dominant congenital cataract has also been reported to be caused by many inherited mutations in γD-crystallin [15]–[33]. γD-crystallin is a monomeirc protein composed of four Greek-key β-sheet structure (Figure 1) [34]. The γD-crystallin mutations may result in inherited cataract by different molecular mechanisms including altering the native structure, decreasing the stability of the native protein, promoting aggregation and/or increasing its sensitivity of UV light. Although most mutations in γD-crystallin are associated with the phenotype of nuclear cataract, the actual mechanism may differ from case to case depending on the role of the mutated residue in γD-crystallin structure and stability and the nature of the substituted residue. Recently, a newly characterized congenital mutation G61C in γD-crystallin has been reported to associate with an irregular phenotype of coralliform cataract [35]. In this research, we studied the effects of the mutation G61C on γD-crystallin structure, stability and aggregation, and the results herein suggested that the decrease in γD-crystallin stability against stresses might contributes to the onset of cataract through protein aggregation.

## Materials and Methods

### Chemicals

Tris, sodium dodecylsulfate (SDS), ultra-pure guanidine hydrochloride (GdnHCl), 1-anilinonaphtalene-8-sulfonate (ANS), thioflavin T (ThT), insulin and bovine serum albumin (BSA) were Sigma products. Dithiothreitol (DTT) was from Promega. All other chemicals were local products of analytical grade.

### Protein expression, purification and sample preparation

The gene of the wild type (WT) human γD-crystallin (*CRYGD*) and the pET-28a expression vector used for site-directed mutagenesis were the same as those described previously [30]. In brief, The WT and mutated proteins were overexpressed in *E. coli* BL21(DE3) Rossetta (Novagen), and a six-His Tag was fused to the N-terminus of the recombinant proteins to facilitate further purification. The expression of the recombinant proteins was induced by the addition of 0.1 mM IPTG. The proteins in the soluble fractions of the *E. coli* ells were collected by affinity chromatography using Ni-NTA resin (Qiagen), and the final products were purified by a Hiload 16/60 Superdex 200 prep grade column or a Superdex G-200 column equipped on an ÄKTA purification system. The protein samples were prepared by dissolving the proteins with a final protein concentration of 0.2 mg/ml in 10 mM phosphate buffered saline (PBS) buffer, pH 7, containing 1 mM EDTA. The protein concentration was determined according to the Bradford method [36].

### Biophysical experiments

The far-UV circular dichroism (CD), intrinsic Trp fluorescence and extrinsic ANS fluorescence spectra were obtained following the procedures described elsewhere [37]. In brief, the CD spectra were collected on a Jasco-715 spectrophotometer using 0.1-cm-pathlength cells. The fluorescence emission spectra were measured on a Hitachi F-4500 spectrofluorimeter using 1-cm-pathlength cuvettes. The Trp fluorescence was measured using an excitation wavelength of 295 nm, while the ANS fluorescence was measured with an excitation wavelength of 380 nm. Before ANS fluorescence measurements, the protein samples were incubated in buffer containing 75-fold ANS for 30 min at 25°C in the dark. The resonance Raleigh light scattering, which is extremely sensitive to the appearance of oligomers that is invisible by turbidity measurements [38], was conducted by measuring the light scattering at 90° using an excitation wavelength of 295 nm. The SEC analysis was performed using a Superdex G-75 column equipped on an ÄKTA purification system. The column was equilibrated with at least 2 column volumes of 10 mM PBS buffer (pH 7.0). All samples were run at a flow rate of 0.5 ml/min at 4°C.

The analysis of the fluorescence data using Parameter A, phase diagram and curve fitting were the same as the procedures described elsewhere [39], [40]. Parameter A, which was obtained through dividing the emission intensity at 365 nm (*I*_365_) by that at 320 nm (*I*_320_), is a sensitive probe to reflect the changes of the shape and position of the intrinsic Trp fluorescence. Phase diagram, which is a powerful tool to detect the potential protein folding intermediate [41], is constructed by plotting *I*_320_ vesus *I*_365_. The fitting of the Trp fluorescence using the discrete states model of Trp fluorophores [42] were performed using a program developed in house [39].

### Protein unfolding induced by GdnHCl

The equilibrium unfolding of the WT and mutated γD-crystallin was performed by incubating the proteins in 10 mM PBS buffer containing various concentrations of GdnHCl at 25°C overnight. The final protein concentration was 0.2 mg/ml. After incubation, the intrinsic fluorescence and turbidity at 400 nm were measured to obtain the unfolding transition curves. The experimental data were fitted to a three-state model using Prism 4.0, and the thermodynamic parameters were obtained by fitting using non-linear least squares regression.

### Protein thermal denaturation

The protein solutions were heated at every given temperature ranging from 25°C to 85°C. The temperature was controlled by a water-bath. After 2 min equilibration, far-UV CD, turbidity, intrinsic and ANS fluorescence measurements were performed to monitor the structural changes of the proteins.

### Acid-induced denaturation

The pH of the protein solutions was adjusted using HCl ranging from pH 2 to pH 8. The protein concentration was 0.2 mg/ml. After 45 min equilibration at room temperature, far-UV CD, intrinsic and ANS fluorescence were measured to reflect the secondary structure, tertiary structure and hydrophobic exposure of the proteins.

### Amyloid-like fibril formation

The formation of the amyloid-like fibrils were performed according to the published procedures [43]. In brief, the proteins were dissolved in 10 mM PBS buffer, pH 3.0, containing 2 mM DTT and 1 mM EDTA. After incubation of 5 mg/ml protein solutions at 37°C for 48 h, the samples were diluted to 0.2 mg/ml for ThT fluorescence measurements. ThT fluorescence was measured by adding 7.5 µM ThT in the samples, and then emission spectra were recorded using an excitation wavelength of 440 nm. For the electron microscopy (EM) experiments, 0.5 mg/ml protein solutions were incubated at 37°C for 72 h. The EM samples were prepared by depositing about 3 µl protein samples onto a freshly glow-discharged carbon coated 300-mesh copper grid followed by negatively staining with 1.25% uranyl acetate for 30 s. The EM experiments were conducted on a Hitachi H-7650B transmission electron microscope.

### Protein aggregation studies

Protein aggregation was monitored by turbidity (absorbance at 400 nm) on an Ultraspec 4300 pro UV/Visible spectrophotometer. Heat-induced protein aggregation was examined by measuring the turbidity of a sample at various temperatures after 10 min equilibration. The time-course aggregation induced by heat was monitored by recording the turbidity data every 2 s for the sample heated at a given temperature. The time-course aggregation during refolding was recorded immediately after the refolding was initiated by diluting the fully denatured proteins into 10 mM PBS buffer in the presence or absence of DTT.

## Results

### The G61C mutation does not affect the secondary and tertiary structures of γD-crystallin

**Figure 2:**
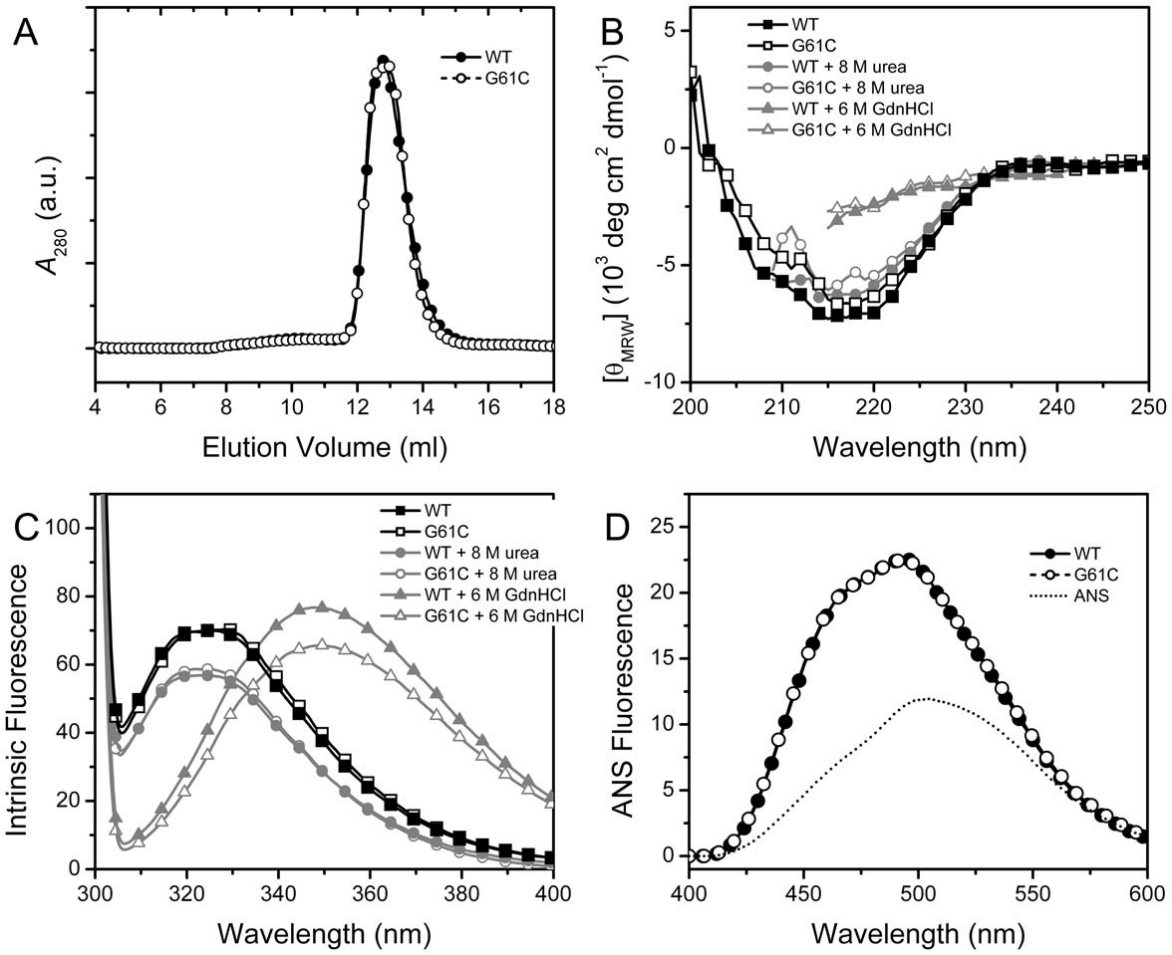
Effect of the G61C mutation on γD-crystallin structure. (A) SEC profile of the WT and mutated γD-crystallin. (B) Far-UV CD spectra of the native proteins and the denatured states in 8 M urea or 6 M GdnHCl. (C) Intrinsic fluorescence spectra of the native proteins and denatured states in 8 M urea or 6 M GdnHCl. The excitation wavelength of the intrinsic fluorescence was 295 nm. (D) ANS fluorescence spectra of the WT and mutated γD-crystallin. The excitation wavelength of the ANS fluorescence was 380 nm. The protein samples were prepared by dissolving the proteins with a final protein concentration of 0.2 mg/ml in 10 mM PBS buffer, pH 7.0, containing 1 mM EDTA and 2 mM DTT.

The purified proteins were found to be homogenous as evaluated by SDS-PAGE and SEC analysis. In the SEC profile, both the WT and mutated proteins eluted as a single peak and the elusion volume was not significantly affected by mutation (Figure 2A). This suggested that the mutation did not affect the oligomeric state or the shape of the γD-crystallin molecule. The effect of the mutation on γD-crystallin structure was investigated via spectroscopic methods (Figure 2B–2D). The CD spectrum of the WT γD-crystallin showed a single negative peak at around 217 nm, revealing that the major regular secondary structure content was β-sheet. This observation is quite consistent with the crystal structure of γD-crystallin [34], which composes four Greek-key β-sheet divided into two domains (Figure 1). The Trp fluorescence of the WT protein was centered at around 325 nm, suggesting that the Trp residues of γD-crystallin mainly located in hydrophobic microenvironments. The hydrophobic exposure of the proteins were evaluated by ANS fluorescence, and no difference was found between the WT and mutated γD-crystallins. The almost superimposed spectra of the WT and mutated proteins shown in Figure 2 clearly indicated that the mutation did not significantly affect the secondary and tertiary structures of γD-crystallin. Both the WT and mutated proteins could maintain most of their native structures in 8 M urea, which can efficiently denature many other proteins [44], suggesting that both proteins were resistant to urea-induced denaturation. In 6 M GdnHCl, both proteins were fully unfolded as revealed by the lack of ordered secondary structures (Figure 2B) and the fully water-exposed Trp residues (Figure 2C).

### The G61C mutation decreases the thermal stability of γD-crystallin

**Figure 3:**
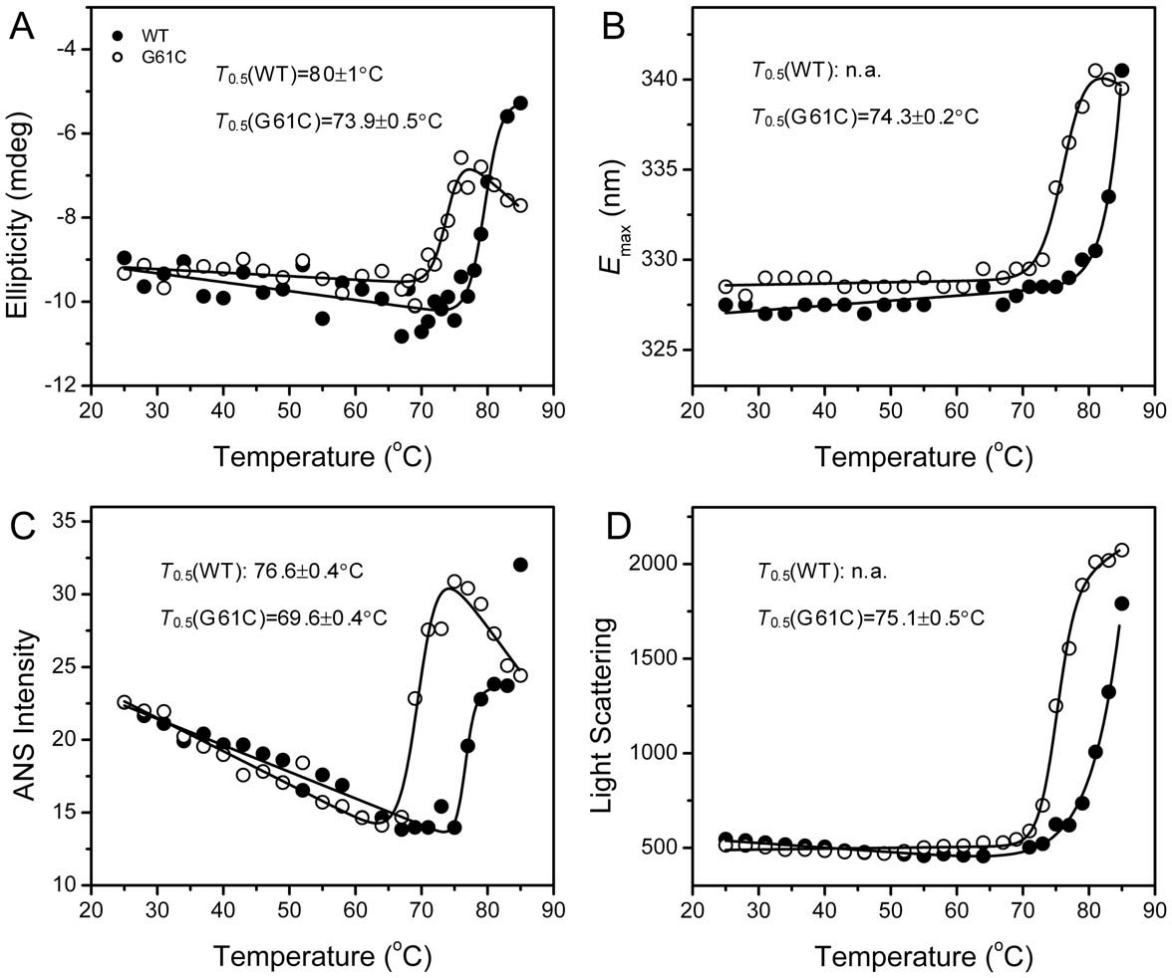
Effect of the G61C mutation on γD-crystallin thermal stability evaluated by the ellipticity at 222 nm in the far-UV CD spectra (A), the maximum emission wavelength (*E*_max_) of the intrinsic fluorescence (B), the intensity at 470 nm of the ANS fluorescence (C) and the resonance Rayleigh light scattering excited at 295 nm (D). The temperature was controlled by a water bath, and the data were recorded at a given temperature after a 2-min equilibration. The raw data were fitted to a two-state model, and the fitting results are shown by solid lines.

The effect of the G61 C mutation on γD-crystallin thermal stability was investigated by increasing the incubation temperature every 2°C from 25°C to 85°C. The structural changes were monitored by far-UV CD, intrinsic fluorescence, ANS fluorescence and resonance Raleigh light scattering, which reflects the secondary structure, tertiary structure or microenvironment of the Trp residues, hydrophobic exposure and oligomeric state of proteins. As presented in Figure 3, all transition curves exhibited an apparent two-state process. It is worth noting that no aggregation was detected when the temperature is below 78°C for G61C and 82°C for the WT γD-crystallin, and the changes in the resonance Raleigh light scattering mainly reflect the occurrence of non-native soluble oligomers [38]. The midpoint temperature where half of the unfolding occurred (*T*_0.5_) of the mutated protein was ∼74°C when probed by far-UV CD, intrinsic fluorescence and light scattering. The *T*_0.5_ value of the WT protein was above 80°C, while the exact value could not be obtained for the data from intrinsic fluorescence and light scattering since the transition curves did not reach its equilibrium. ANS, a fluorescent molecule with the ability to specifically bind with the hydrophobic site [45], is a frequently used probe to detect the hydrophobic exposure of proteins. The *T*_0.5_ value from ANS fluorescence was ∼4°C lower than that from the other three probes for both proteins. Thus the inconsistency of the transition curves suggested that the thermal denaturation of both the WT and mutated γD-crystallin might involve sequential events, and the exposure of the hydrophobic core was an early event during γD-crystallin thermal unfolding as revealed by the ANS fluorescence.

**Figure 4:**
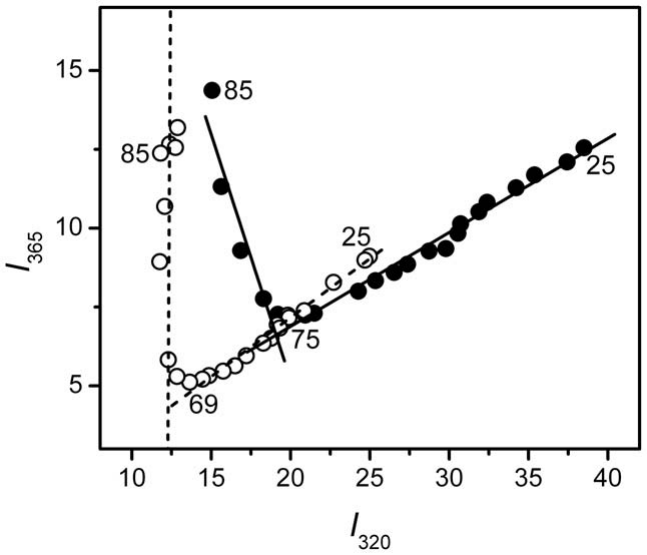
Phase diagram analysis of the intrinsic fluorescence spectra of the WT (filled symbols and solid lines) and mutated γD-crystallin (open symbols and dashed lines) during thermal unfolding. The phase diagram is constructed by monitoring the changes of the fluorescence intensity at 365 nm (*I*_365_) as a function of that at 320 nm (*I*_320_). The temperatures of the starting point, the joint position of the adjacent lines and the end point are labeled.

To further investigate the sequential events occurred during γD-crystallin thermal denaturation, phase diagram was constructed to check whether there existed an unfolding intermediate. In the phase diagram, each linear part indicates a two-state process, and the joint-point of two adjacent lines indicates the appearance of a folding intermediate at this position [41]. As shown in Figure 4, the phase diagram of both the WT and mutated proteins involved two linear parts but different joint positions, confirming that the thermal unfolding of both proteins involved an intermediate: native state (N)→intermediate (I)→aggregates (A).

**Figure 5:**
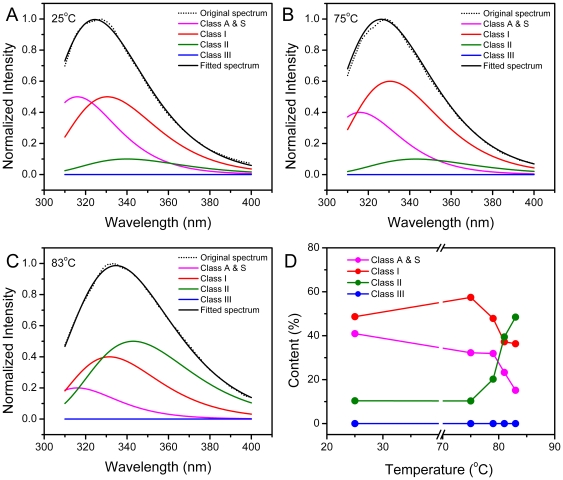
Fitting of the intrinsic fluorescence spectra of the WT γD-crystallin recorded at 25°C (A), 75°C (B) and 83°C (C) by the discrete state model of Trp residues in proteins. The fitted spectra are the sum of the four spectral components: Class A & S centered at ∼318 nm is from the fluorophores in highly hydrophobic and rigid microenvironments, Class I at ∼330 nm reflect buried fluorophores inaccessible to solvent, Class II at ∼340 nm is assigned to fluorophores exposed to bound water, and Class III fluorophores at ∼350 nm are highly exposed to solvent [46]. The experimental data are shown as dotted lines. (D) Thermal dependence of the peak areas of the four fluorophores.

γD-crystallin contains four Trp residues. When fitted by the discrete state model [42], [46], the intrinsic Trp fluorescence spectrum of the native γD-crystallin contains two major fluorophores, the Class A & S and Class I fluorophores, which centered at ∼318 nm and 330 nm, respectively (Figure 5). The peak area ratio was close to 1∶1 for the two fluorophores. The previous mutational analysis indicates that the fluorescence emission is significantly quenched for the W69-only and W157-only mutants, and the emission maximum is centered at 327 nm and 318 nm for the W43-only and W131-only mutants, respectively [47]. Thus it seems that in the fluorescence of native γD-crystallin, the Class A & S fluorophore was mainly contributed by W131, and the Class I fluorophore was mainly by W43. The existence of a minor content of Class II fluorophores centered at 340 nm may arise from the fast exchange of protein conformations in solutions. At 75°C where the intermediate state appeared during the thermal unfolding of the WT γD-crystallin, the contribution of the Class A & S fluorophore decreased, while that of the Class I fluorophore increased correspondingly. This may result from an alteration in the microenvironments of W131 in the intermediate state or an additional contribution by the Trp residues that are quenched in the native state. At temperatures above 75°C, the contents of both the Class A & S and Class I fluorophores decreased, while that of Class II increased continuously. This suggested that the microenvironments of all Trp residues were changed to the more solvent-accessible state upon heating, which is confirmed by the simultaneous increase in the hydrophobic exposure as monitored by ANS fluorescence (Figure 3C). Similar results were obtained for the mutated γD-crystallin except that the transitions occurred at a relatively lower temperature when compared with the WT γD-crystallin.

Thus the results in Figures 3, 4, and 5 suggest that the thermal denaturation of γD-crystallin involved an intermediate state with altered microenvironments around the Trp residues. Further unfolding of this intermediate was accompanied by large hydrophobic exposure. The significant increase of the hydrophobic exposure might directly relate to the aggregation-prone property of the proteins at high temperatures. The G61C mutation did not alter the thermal unfolding pathway of γD-crystallin, but destabilized the protein to reach the intermediate state at a relative low temperature.

### The G61C mutation decreases the structural stability of γD-crystallin against GdnHCl-induced denaturation

**Figure 6:**
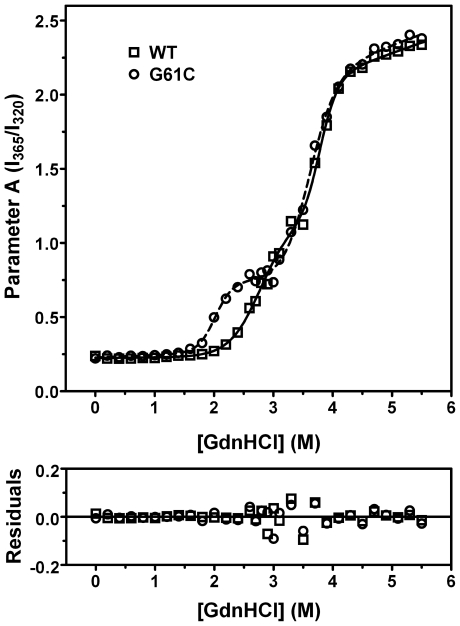
Effect of the G61C mutation on the unfolding of γD-crystallin induced by GdnHCl. Parameter *A* is the ratio of the intensity of the intrinsic fluorescence at 365 nm to that at 320 nm (I_365_/I_320_). The data were fitted to a three-state model, and the fitting results are shown by lines.

Equilibrium unfolding experiments were carried out to probe the effect of the mutation on the structural stability of γD-crystallin against chemical denaturants. Previous studies have shown that the unfolding of γD-crystallin undergoes a multi-state process with an intermediate populated under equilibrium unfolding conditions: native state (N)→intermediate (I)→unfolded state (U) [47], [48]. No off-pathway aggregation was observed when monitored by either turbidity at 400 nm or resonance Raleigh light scattering (data not shown). As presented in Figure 6, the unfolding of both the WT and mutated γD-crystallin was best-fitted by a three-state model when monitored by the intrinsic Trp fluorescence. The G61C mutation did not significantly affect the unfolding pathway of γD-crystallin. However, the midpoint of the N→I transition moved to a lower GdnHCl concentration, while the I→U transition was not significantly affected. Quantitatively, the midpoints of the N→I transition were 2.8 M and 2.0 M, and those of the I→U transition were 3.7 M and 3.6 M for the WT and G61C γD-crystallin, respectively. These results suggested that the G61C mutation significantly decreased the structural stability of the native state of γD-crystallin against chemical denaturants, but had little effect on the properties of the folding intermediate. This conclusion is consistent with the previous observation that the N→I transition of γD-crystallin denaturation induced by GdnHCl involves the unfolding of the N-terminal domain where G61 located [49].

### The G61C mutation does not significantly affect the stability of γD-crystallin against acid-induced unfolding

**Figure 7:**
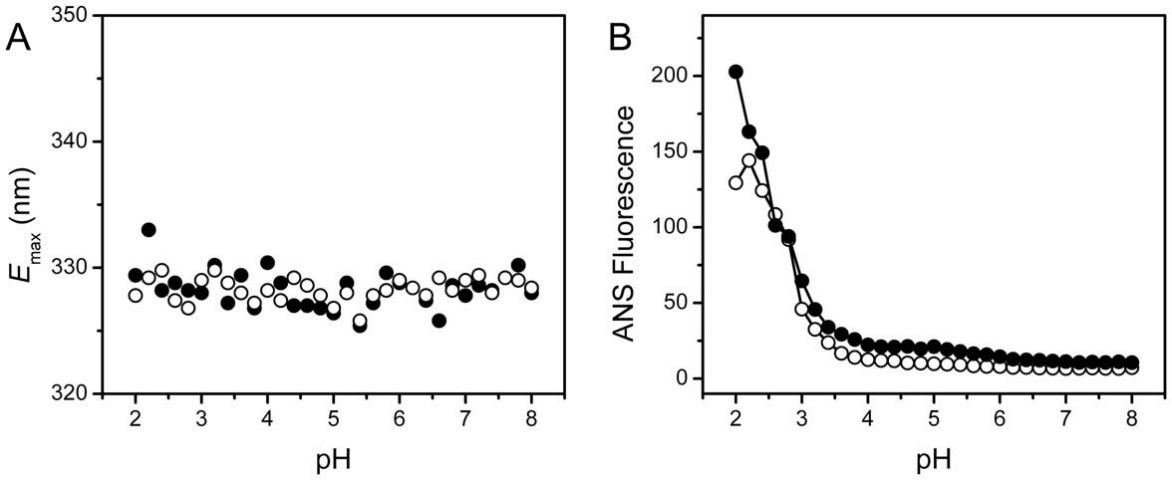
Effect of the G61C mutation on the acid-induced unfolding of γD-crystallin evaluated by the *E*_max_ value of the intrinsic fluorescence (A) and the intensity at 470 nm of the ANS fluorescence (B).

Acidosis is a frequently encountered stress in bodies. The acid resistance of the WT and mutated γD-crystallin was evaluated by measuring the structural changes when decreasing the pH of the solutions. No significant changes in either the secondary or tertiary structures were observed for both proteins as reflected by the almost constant CD signal (data not shown) and intrinsic Trp fluorescence (Figure 7). An increase in the ANS fluorescence was observed for both proteins at pH below 3.5. However, the mutation did not significantly affect the transition curve from ANS fluorescence. These results indicated that the mutation did not alter the acid-resistant property of γD-crystallin.

### The G61C mutation promotes the fibrilization of γD-crystallin

**Figure 8:**
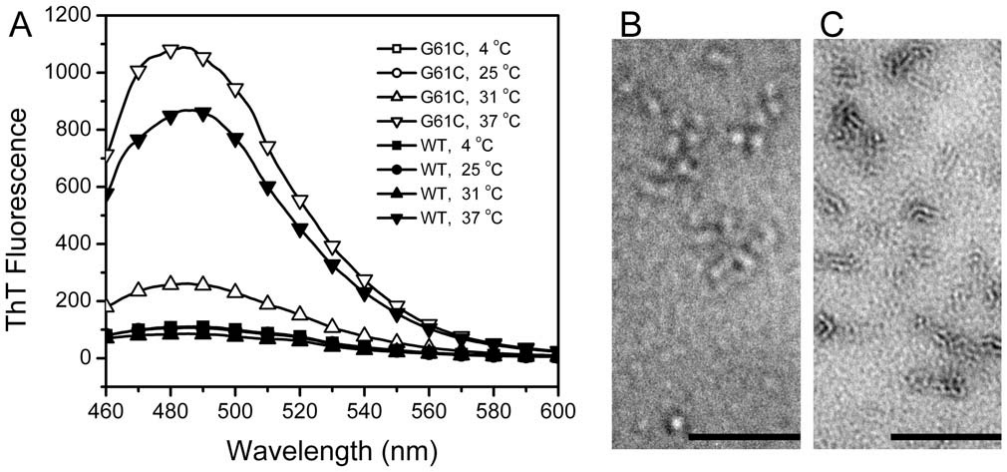
Effect of the G61C mutation on the fibrilization of γD-crystallin under acidic condition. (A) ThT fluorescence of the WT and mutated proteins in 10 mM PBS buffer, pH 3, incubated for 48 h at 4°C, 25°C, 31°C and 37°C. The protein concentration was 5 mg/ml. The spectra recorded at 4°C or 25°C superimposed each other for the WT and mutated proteins. (B and C) The transmission EM picture of 0.5 mg/ml WT (B) and G61C γD-crystallin (C) samples incubated at 37°C for 72 h. The bar represents 200 nm.

Cataract is a disease caused by protein aggregation. To further study the effect of the mutation on γD-crystallin fibrilization, the WT and mutated proteins were incubated at a high protein concentration (5 mg/ml) at acidic conditions, and then ThT fluorescence was used to probe the existence of amyloid-like fibrils. As presented in Figure 8, neither the WT nor the mutated protein formed fibrils at temperatures below 25°C. At 31°C, the ThT fluorescence of the mutated protein was significantly increased, while that of the WT protein did not. At body temperature, the ThT fluorescence of the mutant was much higher than that of the WT protein. The fibrilization of the proteins was also visualized by transmission EM. At a low protein concentration of 0.5 mg/ml, the mutant was found to convert to considerable amounts of amyloid-like fibrils, whereas the WT protein was at the initial stage of aggregation and formed bead-like structures. These observations suggested that the mutant was more prone to form amyloid-like fibrils than the WT protein when subject to certain physiological stresses or pathological conditions.

### The addition of DTT does not significantly affect the thermal aggregation of γD-crystallin

**Figure 9:**
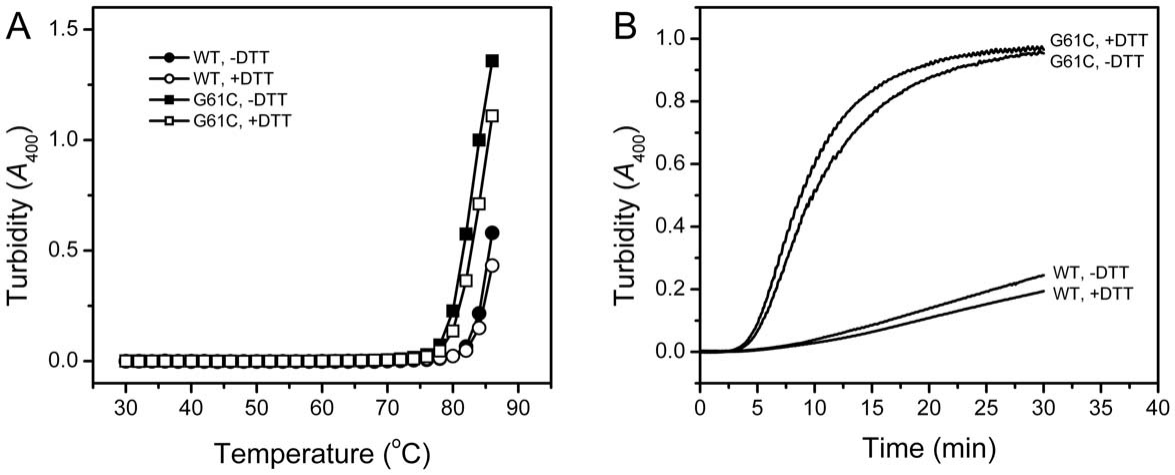
Effect of DTT on γD-crystallin thermal aggregation. (A) Temperature-dependence of γD-crystallin thermal aggregation monitored by turbidity. (B) Time-course aggregation of γD-crystallin at 80°C. The samples were prepared in 10 mM PBS buffer, pH 7, with or without the addition of 2 mM DTT.

A previous study has shown that one cataract-linked mutation R14C introduces an additional reactive thiol group, which contributes to the intermolecular cross-link of the γD-crystallin molecules and leads to aggregation therefore [50]. To investigate whether the cataract caused by the G61C mutation followed this molecular mechanism, we studied the effect of DTT on the thermal aggregation behavior of the WT and G61C γD-crystallin (Figure 9). At temperatures above 80°C, although the addition of DTT could decrease the turbidity at each given temperature, the whole process was not significantly affected. To avoid errors raised from the temperature adjustment, time-course aggregation was monitored by heating the protein solutions continuously at 80°C. As shown in Figure 9B, no significant changes were observed between the samples with and without the addition of DTT. Similarly, no significant effect of DTT was also observed for γD-crystallin aggregation induced by dilution-initialed refolding and acid-induced fibrilization (data not shown). These observations suggested that the reactive –SH group was not significantly altered by the G61C mutation and that the intermolecular disulfide formation was not a major cause of γD-crystallin aggregation.

## Discussion

γD-crystallin is one of the major structural proteins in human eye lens. The solubility and stability of γD-crystallin play a crucial role in maintaining the optical properties of the lens during the life span of an individual. Up to now, 19 mutations in γD-crystallin have been reported to be associated with the occurrence of autosomal dominant congenital cataract [15]–[33], implying that γD-crystallin plays a significant structural role in maintaining the transparency of the lens.

Previous studies revealed that these mutations may affect γD-crystallin via different molecular mechanisms. One specific group of mutations is nonsense and frame-shift mutations, which significantly altered the primary structure of the protein. Particularly, the truncation usually appears at the C-terminus of γD-crystallin, and the truncated mutants are not able to fold correctly to a native-like soluble form when overexpressed in *E. coli* (Cai and Yan, unpublished data). It seems that the inability to fold into soluble proteins is the dominant mechanism of congenital cataract caused by truncations. Among the cataract-linked γD-crystallin mutations, the most frequently occurred mutation is the substitution of a charged residue to non-charged residue or vice versa (R14C, R36S, W43R, R58H, E107A and R140X), which may decrease the solubility of γD-crystallin by simply modifying the surface charge distributions. Actually, the surface charge-altering mutation has also been frequently characterized in the other crystallins [8]. Some mutations may affect solubility by modifying the structure of γD-crystallin. It has been reported that the P23T mutation increases the surface hydrophobicity and lowers γD-crystallin solubility thereby [51]. The W43R mutation also leads to a significant increase in the hydrophobic exposure when compared to the WT protein [30]. The W43R mutation has also been found to have deleterious effects on γD-crystallin structure, thermal stability and resistance to UV light stress. In summary, up to now, the mutations in γD-crystallin may lead to cataract by altering the native structure/surface properties, decreasing the stability, promoting aggregation and/or increasing its sensitivity against the UV irradiation.

In this research, we found that the inherited mutation G61C did not significantly modify the native structure of γD-crystallin as revealed by spectroscopic experiments. G61 is located at the last strand of the second Greek-key motif in the N-terminal domain, and the G61C mutation seems to have little impact on the fold of the motif. The little changes in the native structure with no additional hydrophobic exposure suggested that the mutation might not significantly interfere with the correct protein-protein interaction network in the normal conditions of the lens, unlike what has been observed for the E107A mutation in γD-crystallin [52] as well as the mutations in the other crystallins [52]–[54].

The alteration of the number of Cys residues in a protein may affect its responses against redox disturbance in the cells. The WT γD-crystallin contains six Cys residues, and only one is exposed to the solvent [50]. The detailed study in the R14C mutation indicates that the introduction of an extra reactive thiol group results in the formation of intermolecular disulfide bond, which further alters the solvent-protein interactions [50]. However, we found that the addition of DTT had little effect on the aggregation of both the WT γD-crystallin and the G61C mutant, suggesting that intermolecular disulfide cross-link contributed little to the aggregation of both proteins.

The results herein suggest that the decreased stability of γD-crystallin by the G61C mutation was the major cause of the onset of hereditary cataract. We found that the mutation significantly decreased γD-crystallin stability against heat and chemical denaturants. The GdnHCl-induced unfolding of γD-crystallin has been well characterized to be a three-state process involved an intermediate with an unfolded N-terminal domain and a folded C-terminal domain [47], [48]. In this research, we found the thermal unfolding of γD-crystallin is also a three-state process. However, unlike the intermediate appeared in GdnHCl-induced unfolding, the intermediate in γD-crystallin thermal unfolding maintained most of its native structure but an alternation of the hydrophobic core around the Trp residues (Figure 5). The unfolding of the intermediate led to a further disruption of the native structure of γD-crystallin, which increased the surface hydrophobicity of the molecules and resulted in serious aggregation. For both three-state unfolding processes, the mutation did not alter the unfolding pathway of γD-crystallin, but affected the N→I transition. This means that the mutation destabilized the native structure, but had no significant effect on the properties of the intermediates. This conclusion is consistent with the fact that G61 is located at the surface of the N-terminal domain and contributes little to the overall fold of the Greek-key motif (Figure 1).

Unlike the destabilization effect on heat- and GdnHCl-induced denaturation, the G61C mutation did not affect the acid-induced unfolding of γD-crystallin. However, the mutant was more prone to form amyloid-like fibrils than the WT when incubated at temperatures above 30°C. It is worth noting that the denaturation of proteins is temperature-dependent. Thus although the mutation had no effect on the acidosis resistance of γD-crystallin, a combination of both acidosis and heat stress could promote the unfolding and aggregation of the mutated protein. At a low concentration of 0.5 mg/ml, the mutant was found to successfully convert to mature fibrils after 72 h incubation, while the WT protein was still at the initial stage of fibrilization. These observations suggested that although the *in vitro* system showed that the aggregation of the mutant required a relative high temperature (above 75°C), the proteins might be more prone to aggregate under relatively mild conditions when the lens cells suffered a combination of various stresses during development.
